# Editorial: The Search for Biomarkers in Psychiatry

**DOI:** 10.3389/fpsyt.2021.720411

**Published:** 2021-07-12

**Authors:** Maria S. Garcia-Gutierrez, Jorge Manzanares, Francisco Navarrete

**Affiliations:** Instituto de Neurociencias, Universidad Miguel Hernández-CSIC, Alicante, Spain

**Keywords:** biomarkers, central biomarkers, peripheral biomarkers, omics, psychiatry

Psychiatric disorders present a high level of complexity in many aspects. Contrary to other diseases, they are classified by diagnostic categories with a broad variety list of symptoms. This categorical organization results in a great clinical heterogeneity among patients diagnosed from the same psychiatric illness. Besides, the high rate of comorbidity among psychiatric disorders is an additional distinguishing factor that makes diagnosis complicated. On the top of that, the limited knowledge of the molecular mechanisms underlying mental disorders, greatly contribute to the reduced efficacy of current pharmacological treatments, being especially poor as the severity of the disease increases. This clinical situation stimulated the searching of biomarkers to improve prevention, diagnosis and treatment of psychiatric disorders. This special issue includes one review article of García-Gutiérrez et al., summarizing the concept and types of biomarkers in psychiatry and providing examples about the most promising results achieved sorted by categories (genetics, transcriptomics, proteomics, metabolomics, and epigenetics). The review includes a final conclusion that remarks the future challenges required to reach the goal of developing valid, reliable and broadly-usable biomarkers for psychiatric disorders and their treatment. Complementary, the opinion article of Vinberg further explored the main limitations and the future perspectives in the searching of peripheral biomarkers in psychiatry.

The potential role of key targets of the immune and endocannabinoid systems are studied in detail in three articles. The brief research report of Larsen et al. provide evidences about an association between alterations in different cytokines and motor activity in an acute psychiatric population, suggesting that some cytokines deserve further exploration as biomarkers for predicting and treating changes in motor activity. Additionally, the review article of Momtazmanesh et al., is a fantastic review summarizing the main cytokine alterations observed in schizophrenic patients, their potential link with certain symptoms and their potential clinical impact. Similarly, the review article of Navarrete et al., provide the most promising results achieved to date that support the potential role of different targets of the endocannabinoid system as biomarkers in several psychiatric conditions.

Besides, the review of Li Z. et al., is an excellent article containing the most recent studies supporting the potential use of circular RNA as biomarkers in two major psychiatric disorders, depression and schizophrenia. Finally, the review of Jurado-Barba et al., covers the potential clinical use of EEG as clinical assessment in alcohol dependence.

A total of seven articles are focused on the identification of biomarkers in major depressive disorder (MDD). Zhao et al., analyse the utility of CACNA1C rs1006737 polymorphism together with exposure to threatening life events as predictive biomarkers for MDD, pointing out this polymorphism as a target for new pharmacological treatments in this psychiatric disorder. Explore the use of high heart rate as biomarker for assessing the outcome of reinstatement at work in MDD patients who took a leave of absence, with promising results. Zhao et al., carried out the study of different single nucleotide polymorphisms of FoxO1, A2M, and TGF-B1 in MDD patients, demonstrating a potential association between some of them and the environment in MDD. Authors suggest that these SNPs may be useful for assessing MDD risk improving prevention.

An emerging type of biomarkers is metabolomic alterations. In this respect, Gao et al., focus on studying the role of 36 metabolic biomarkers in depression. The research sheds light on potential important metabolites and enzymes in the underlying molecular mechanisms of depression.

Troyan and Levada, wrote an exciting original article revealing the correlation between serum concentrations of the neurotrophin BDNF and IGF-1 and MDD. Interestingly, changes in these targets are also observed after 8 weeks of treatment with the antidepressant vortioxetine, supporting that both may be useful as biomarkers of diagnosis and treatment outcome in MDD. Similarly, Köhler-Forsberg et al., provide the results of an open label clinical trial in MDD patients supporting the utility of a panel of biomarkers (serotonin 4 receptor PET brain imaging, fMRI, cognitive-, EEG-and peripheral biomarkers) for MDD diagnosis and for predicting pharmacological efficacy. Ho et al., presented a comprehensive update regarding functional near-infrared spectroscopy (fNIRS) as a biomarker for guiding diagnosis and monitoring treatment response in MDD.

Kittel-Schneider et al., covered the emerging role of proteomics profile as a diagnostic tool for distinguish bipolar from unipolar depression. The results support the potential role of the multivariate predictive model proposed by authors as a predictive biomarker model useful to discriminate between these psychiatric entities.

Interestingly, Cross et al., presented a case report suggesting the clinical relevance to discard the presence of infectious diseases, such as Lyme borreliosis or PANDAS, in patients presenting neuropsychiatric symptoms. These results data, although preliminary, support the close connection between the immune system and the brain.

The original article of Li G. et al., point out lower serum uric acid concentrations as a biomarker for predicting depression in stroke patients. These interesting results support the development of additional longitudinal and clinical studies.

Taken together, these articles updated new advances about peripheral and central biomarkers using a variety of methods (proteomics, transcriptomics, genetics, and imaging) in psychiatric clinical conditions and samples to identify alterations with specific traits of the disease or with the outcome of the pharmacological treatments.

## Author Contributions

All authors listed have made a substantial, direct and intellectual contribution to the work, and approved it for publication.

## Conflict of Interest

The authors declare that the research was conducted in the absence of any commercial or financial relationships that could be construed as a potential conflict of interest.

